# Exploring the Icarus Paradox in Indonesia's Specialist Medical Education System Using the Public Perspective From Online Media: Convergent Mixed Methods Study

**DOI:** 10.2196/60452

**Published:** 2026-01-26

**Authors:** Faisal Binsar, Mohammad Hamsal

**Affiliations:** 1 Management Department Binus Online Learning Binus University Jakarta, Jakarta Indonesia; 2 Binus University Binus Business School Doctor of Research in Management Binus University Jakarta, Jakarta Indonesia

**Keywords:** Icarus Paradox, aspirations, realities, medical education, student well-being, community perspective, online reviews

## Abstract

**Background:**

The Icarus Paradox in health care refers to the tension between the ambition to succeed as a specialist doctor and the limitations of the medical education system. Indonesia aspires to produce quality doctors, yet limited infrastructure and resources hinder the educational journey of prospective specialists.

**Objective:**

This study aimed to identify the Icarus Paradox in Indonesia's specialist medical education by examining prospective specialist medical students and the quality of health services and by analyzing how this paradox is reflected in society’s perspectives.

**Methods:**

Using a convergent mixed methods design, this study integrated quantitative content analysis of 5047 online reviews across multiple platforms with qualitative thematic and cognitive analysis using NVivo 14, combining sentiment classification and topic coding.

**Results:**

Twitter contributed 573 (11.3%) of 5047 reviews, with 218 (38%) negative, 251 (43.8%) neutral, and 104 (18.2%) positive entries. TikTok generated 282 (5.6%) reviews, the majority being neutral (n=225, 79.5%). YouTube produced 96 (1.9%) reviews, with 89 (92.7%) neutral entries. News platforms exhibited the largest volume (n=3040, 60.2%) of reviews, with 2885 (94.9%) neutral, 105 (3.5%) positive, and 50 (1.6%) negative entries. Blogs and websites contributed 353 (7%) and 692 (11.3%) reviews, respectively, with neutral sentiment dominating (n=329, 93.2%, for blogs and n=599, 86.6%, for websites). Three cognitive perspectives demonstrated the Icarus Paradox in the Indonesian medical education system: education system, society’s views of students, and health care services. Although there are aspirations to improve education and health care quality, these ambitions often collide with structural challenges, such as resource shortages, heavy workloads, and limited accessibility, which link directly to cognitive themes of stress, resilience, and ethical dilemmas. We proposed a conceptual model to illustrate these dynamics.

**Conclusions:**

Our findings offer insights into the Icarus Paradox in Indonesia’s medical education system, highlighting its complexity and reinforcing the need for systemic reform. Beyond academic relevance, the findings also emphasize the importance of strengthening student mental health support, ensuring equitable access to health care, and enhancing regulatory oversight of training. This was not a clinical trial. Although limited by reliance on online reviews, the results underscore the urgent need for targeted policy interventions in medical education and health care services.

**Trial Registration:**

ClinicalTrials.gov registration: NCT123456

## Introduction

### Background

The Icarus Paradox, originally used in management studies to explain how strengths can become liabilities, offers a useful lens to examine paradoxical dynamics in specialist medical education [1]. Although such programs are designed to produce highly competent professionals, they often generate conditions that undermine performance and well-being. These paradoxes arise from three interconnected sources.

First, systemic educational design—marked by rigid curricula, competitive entry pathways, and limited infrastructure—restricts flexibility and adaptability. Second, entrenched institutional cultures characterized by hierarchy, conformity, and normalization of bullying shape learning environments where silence is often preferred over expression [2-6]. Third, the expectation that trainees endure prolonged working hours, intensive workloads, and psychological pressure with minimal support places substantial demands on individual resilience. These interactions illustrate how a system striving for excellence may inadvertently create vulnerabilities that undermine its goals.

Paradox theory underpins this study, emphasizing the simultaneous coexistence of mutually contradictory yet interdependent elements that persist over time [7,8]. A paradox is defined as “contradictory yet interrelated elements that exist simultaneously and persist over time” [9]. In the context of specialist medical education, these tensions emerge when academic rigor, resilience, and institutional pride—typically considered strengths—lead to burnout, intimidation, or compromised well-being.

Medical students and residents frequently face extreme academic pressure that may lead to burnout or depression [10]. Burnout is characterized by emotional exhaustion, depersonalization, and reduced personal accomplishment [11], while depression comprises persistent sadness, loss of interest, or suicidal ideation. Although overlapping, the two conditions are conceptually distinct. Resident doctors are particularly vulnerable to both, and their compromised well-being negatively affects patient care quality [11]. Media coverage frequently portrays emergency conditions in specialist medical education, illustrating the severity of burnout and distress among trainees [12-14]. Policymakers have expressed growing concern over declining patient care quality linked to these systemic issues [15].

**Figure 1:**
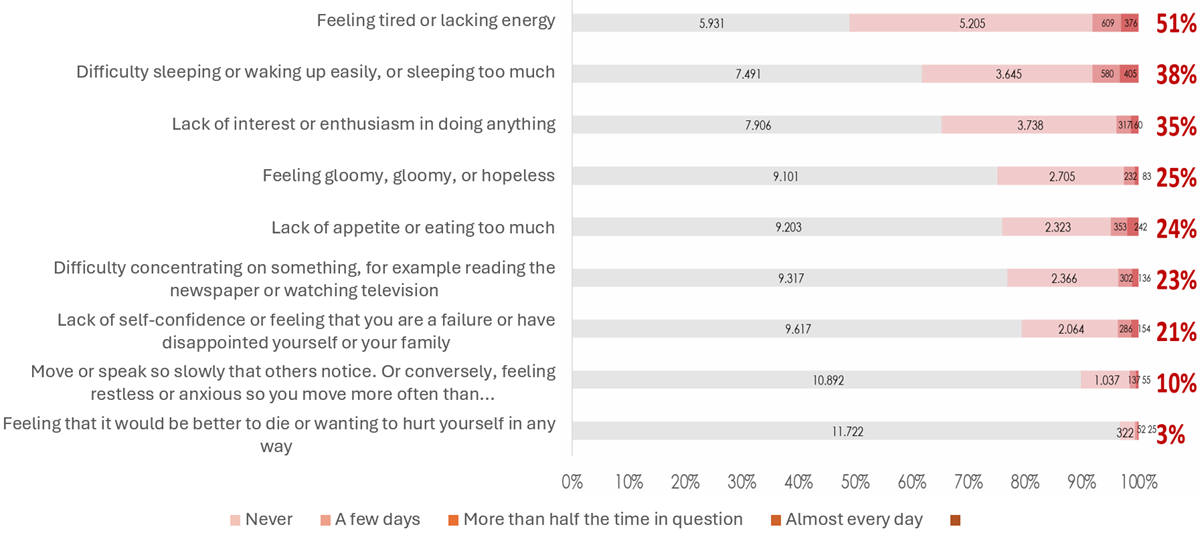
PPDS mental health screening at vertical education hospitals. PPDS: *Program Pendidikan Dokter Spesialis* (Specialist Doctor Education Program).

A national survey conducted by the Ministry of Health of Indonesia in 2024 reported that among 12,121 participants in the *Program Pendidikan Dokter Spesialis* (PPDS; Specialist Doctor Education Program) at 28 vertical education hospitals, 2716 (22.4%) showed depressive symptoms [16]. Additionally, 6182 (51%) reported fatigue or low energy, 4606 (38%) experienced sleep disturbances, 4242 (35%) reported reduced interest in daily activities, and 400 (3.3%) indicated suicidal ideation or self-harm thoughts (Figure 1) [16]. These results underscore the significant mental health challenges facing trainees.

The same survey revealed that the specialties with the highest depressive symptoms were pediatrics, with 1672 (13.8%) trainees; orthopedics and traumatology, with 1491 (12.3%) trainees; and internal medicine, with 1091 (9.0%) trainees [16]. This situation raises concerns regarding both trainee well-being and patient care quality. Media reports frequently highlight psychological crises among specialist medical trainees, echoing public worries about inadequate institutional support. High academic expectations combined with insufficient psychosocial resources leave many trainees struggling with anxiety, exhaustion, and a profound sense of entrapment.

These challenges reflect worldwide patterns. In the United States, a prospective study found that depressive symptoms increase progressively across medical training, with fourth-year students having more than eight times the odds of emotional exhaustion compared to first-year students [17]. More than one-third of US family medicine residents report burnout, particularly women and those with high educational debt [18]. In Canada, over half of resident doctors experience burnout, especially those working over 80 hours per week [19]. A scoping review reported a wide variation in the prevalence of burnout and depression among physicians and residents, with the evidence consistently indicating greater vulnerability among women [20].

Regional data reveal similar patterns: Iranian medical students experience significant burnout associated with academic pressures [21]; Hong Kong students commonly report burnout linked to poor sleep and limited exercise [22]; and Thai clinical students report high emotional exhaustion and depersonalization, particularly among males and those with lower academic performance [23]. The British Medical Association highlights excessive demands and poor working conditions as major contributors to declining physician well-being worldwide [24].

Although these issues occur worldwide, Indonesia’s case is particularly severe due to rigid systemic design, hierarchical cultures, and limited psychosocial support. These characteristics intensify paradoxical tensions consistent with broader paradox scholarship, which argues that unresolved tensions often generate both innovation and dysfunction [7,8].

### Aims of the Study

This study had two primary aims. Theoretically, it used the Icarus Paradox to explain how exclusivity and academic excellence within Indonesia’s specialist medical education system can inadvertently produce negative outcomes for trainee well-being and service quality. Practically, it examined public perspectives captured through online media to identify institutional gaps in responsiveness to societal expectations, health care market needs, regulatory dynamics [25-28], and medical technology advancement [29]. In doing so, the study asked how the Icarus Paradox manifests within Indonesia’s specialist medical education system from the viewpoint of the public, and how these insights can guide policymakers and training institutions toward reforms that balance quality, responsiveness, and trainee well-being [29].

### Theoretical Background

Paradoxical dynamics have become increasingly prominent in contemporary organizations as scholars examine how contradictory yet interrelated elements coexist and shape behavior over time. Such tensions surface when organizations must respond to opposing demands that are both legitimate, generating cycles of alignment, resistance, and adaptation. These dynamics appear across institutional logics, identity structures, and organizational processes, with senior leaders experiencing them as strategic contradictions and employees encountering them in daily routines and socioemotional relationships [30]. Although paradox scholarship has generated numerous influential studies [31-33], its rapid adoption also raises concerns about conceptual stretching, overconfidence in prevailing interpretations, and labeling practices that may reinforce dominant theoretical positions [34].

This study adopted the Icarus Paradox as its core theoretical lens. Rooted in Greek mythology, the metaphor describes how Icarus’s success in flying ultimately led to failure when he ignored warnings and flew too close to the sun [35-37]. In strategic management, the Icarus Paradox explains how organizations’ strengths can become vulnerabilities when past success fosters overconfidence, rigidity, or reluctance to adapt [35,38]. Institutions that excel may inadvertently undermine their own survival by persisting with established routines despite environmental shifts [39]. This phenomenon, often referred to as “vicious inertia,” reflects a broader tension between stability and adaptation—an enduring hallmark of paradox theory. Comparable dynamics emerge in education, where aspirations for excellence collide with resource scarcity, rigid structures, and uneven institutional preparedness [40].

Paradoxical tensions are also evident in medical education. High academic standards and professional expectations are essential for producing competent physicians, yet they simultaneously generate psychological burden among trainees. Medical students frequently report stress, burnout, and depression [41,42], and Indonesia’s specialist medical trainees experience particularly elevated levels of distress, with consequences for well-being and professional performance [10]. These tensions represent *strategic paradoxes*—institutions prioritizing excellence and productivity at the expense of trainee mental health. Identity paradoxes also emerge as students’ aspirations to become empathetic, capable physicians conflict with the exhaustion, self-doubt, and emotional strain encountered during training. Institutional logic paradoxes further arise when cultures valorize endurance and sacrifice, while health systems increasingly demand empathy, patient-centeredness, and clinician well-being.

These psychological strains carry serious implications. Burnout and depressive symptoms reduce clinical performance, increase the likelihood of errors, and diminish empathy toward patients [43]. If unaddressed, these conditions often persist into residency and professional practice, reinforcing negative cycles within the health care workforce [11]. Medical education paradoxes therefore represent tangible, lived realities that directly influence patient safety and service quality.

Addressing these tensions requires intentional institutional support. Access to mental health services, resilience education, and curricula integrating well-being are foundational [42,43]. Another paradox emerges from the gap between expected graduate competencies and actual system-level outcomes: training aspires to produce highly capable specialists, but health care performance does not always match societal expectations [44]. Continuous reform is therefore essential to align educational structures with evolving health care needs, while recognizing human limitations.

Social media has recently played a dual role in illuminating these paradoxes. On the one hand, it facilitates collaboration [27], resource sharing [45], knowledge exchange [46], and peer support that strengthens students’ sense of belonging [26,47,48]. On the other hand, it can propagate misinformation, amplify stress, and distract from academic focus [49]. Online engagement also provides a space for public commentary [50,51], revealing lived experiences of medical trainees and societal expectations of the health care system. Thus, social media functions both as a catalyst and a mirror of the paradoxes embedded within specialist medical education.

## Methods

### Study Design

This study was registered at ClinicalTrials.gov (NCT123456). The research used a convergent mixed methods design [52], in which quantitative and qualitative approaches were conducted simultaneously and coalesced to provide a rich understanding of the Icarus Paradox within the specialist medical trainee education system in Indonesia. In this design, quantitative sentiment analysis and qualitative thematic analysis were conducted independently but merged at the interpretation stage to facilitate triangulation of findings.

### Data Collection

Data collection was carried out over 30 days (March 24-April 23, 2024) using Brand24 [50,53,54], a social listening and monitoring tool that enables focused and comprehensive analysis of conversations on platforms such as Twitter [55], online news portals, blogs, videos, discussion forums, and websites [56,57]. Brand24 was selected over other monitoring tools because it provides reliable access to Indonesian language data, allows automated tracking of multiple keywords, and has been previously validated in academic studies [50].

The search was conducted using a set of keywords relevant to Indonesia’s specialist medical education system, including “PPDS,” “resident doctor,” “specialist candidate,” “collegium,” “medical student,” and “medical education.” The data collected were stored in Microsoft Excel format, containing metadata, such as the posting time, ID or username, source, review content, sentiment category, number of replies, number of likes, number of reposts, number of followers, and influence score.

To ensure the relevance of the dataset, irrelevant or duplicate content was systematically removed. Posts were excluded if they were (1) purely promotional or advertisements, (2) unrelated to medical education despite containing a keyword, (3) spam or bot-generated content, or (4) duplicates of previously captured entries. This filtering step ensured that only meaningful and contextually relevant discussions were retained for analysis.

### Data Analysis

The analysis process involved both quantitative sentiment analysis and qualitative thematic analysis.

For quantitative sentiment analysis, Python was used with text mining and natural language processing (NLP) libraries, such as *NLTK*, *spaCy*, *Pandas*, and *Scikit-learn* [58,59]. Sentiment classification grouped posts into three categories: positive, neutral, and hostile. Additional preprocessing steps included removing hashtags, emojis, hyperlinks, and user mentions, as these elements did not add meaningful semantic value for sentiment classification. The output of this analysis included statistics, graphs, proportions, and trends.

Since the dataset was originally in Indonesian, translation into English was necessary for subsequent qualitative coding. Translation was performed using Google Translate, combined with manual verification by bilingual members of the research team to ensure contextual and semantic accuracy. This step reduced the risk of misinterpretation and enhanced the reliability of findings.

For qualitative thematic analysis, data were imported into NVivo 14 [60]. Coding was performed using a hybrid approach: inductive coding allowed themes to emerge directly from the data, while deductive coding was informed by paradox theory and the Icarus Paradox framework. To enhance intercoder reliability, multiple researchers were involved in the coding process, and discrepancies were resolved through discussion until a consensus was reached. Codes with similar content were grouped into broader themes to identify dominant narratives.

### Integration of Findings

**Figure 2:**
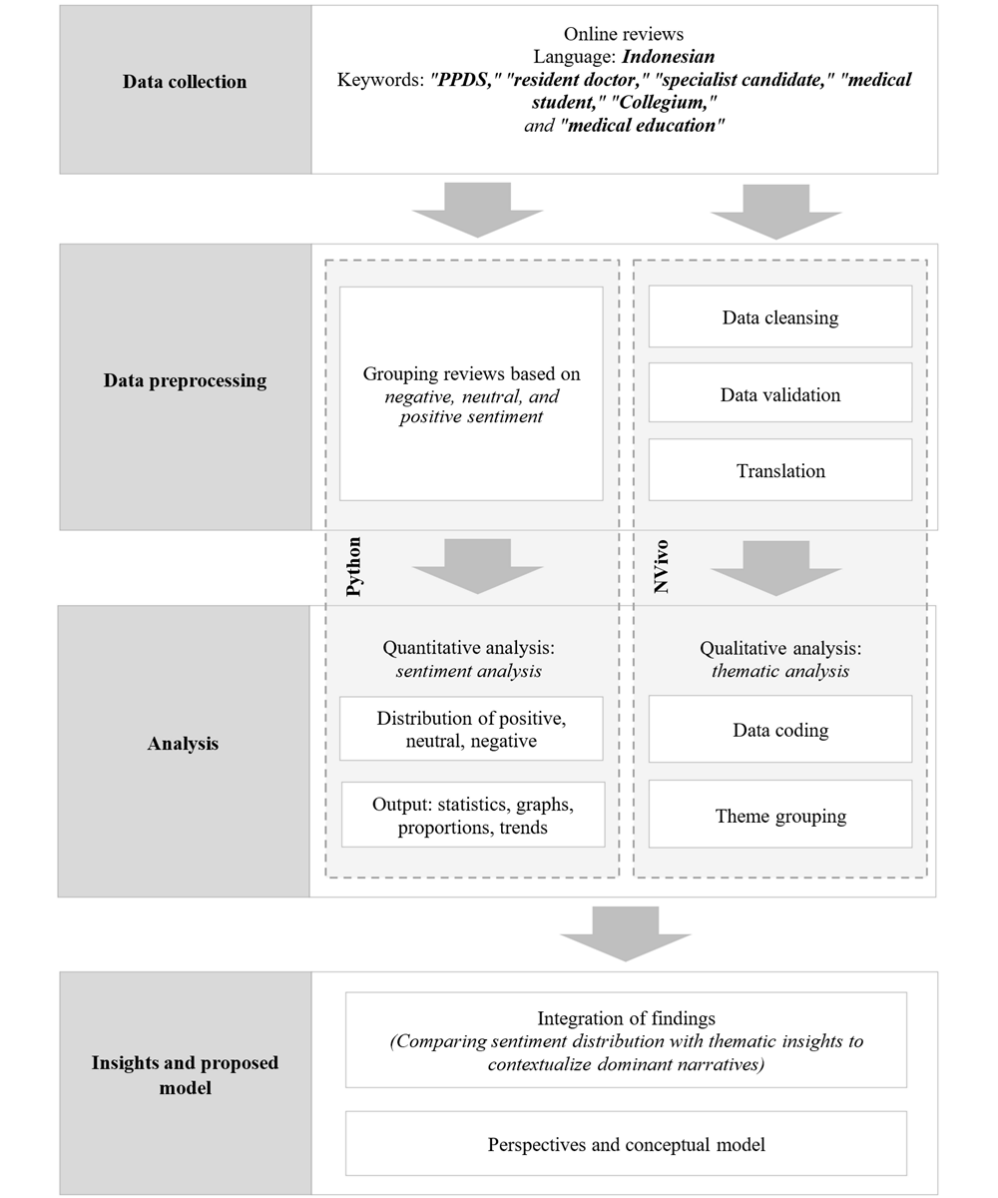
Convergent mixed methods study design. PPDS: *Program Pendidikan Dokter Spesialis* (Specialist Doctor Education Program).

The final stage involved integrating the results from both strands of analysis. Quantitative sentiment trends were compared with qualitative thematic insights to contextualize dominant narratives about specialist medical education. This integration enabled the development of a perspectives-based conceptual model, summarizing how the Icarus Paradox manifests in Indonesia’s specialist medical education system and how public perceptions highlight areas requiring reform. A summary of all stages of the method is shown in Figure 2.

### Ethical Considerations

This study used publicly available, nonidentifiable online data and did not involve interaction with human participants; therefore, Institutional Review Board (IRB) approval was not required. The authors adhered to applicable ethical standards for research using public online data and removed any potentially identifying information during analysis.

## Results

### Online Review Data

Data were collected for 30 days (March 24-April 23, 2024) using Brand24 as the primary social listening and analytics platform [50,53,54]. This tool was selected because of its ability to capture Indonesian language data across multiple platforms, provide sentiment classification, and export structured datasets for further quantitative and qualitative analyses. In total, 5047 reviews were retrieved based on the specified keywords, distributed across digital platforms such as Twitter (X), TikTok, video-sharing sites, online news portals, podcasts, forums, blogs, and websites.

The platform with the highest number of reviews was news portals, with 3040 (60.2%) reviews; followed by websites, with 692 (13.7%), Twitter with 573 (11.3%), and blogs with 353 (7%) reviews. TikTok and video systems additionally contributed significantly, with 282 (5.6%) reviews and 96 (1.9%) opinions, respectively. Podcasts accounted for the best 6 (0.1%) evaluations, and forums contributed 5 (0.1%) reviews, making them the least represented platforms. These percentages provide a clearer picture of the dominance of such structures, with news portals rising to become the number one space for public discourse on professional clinical education in Indonesia. The variation highlights how exclusive audiences use more than one online channel to share their perspectives and worries, underscoring the subject’s relevance in numerous segments of digital media.

### Sentiment Analysis

Sentiment classification of the collected data was conducted using the Brand24 platform [61]. Brand24 integrates artificial intelligence (AI)–driven deep learning with rule-based lexicon techniques, enabling both high accuracy and methodological transparency. Its multilingual sentiment model supports analysis in more than 100 languages, including Indonesian, and its performance has been validated using the *F*_1_-score metric. Based on evaluations of 50,000 mentions, overall accuracy increased [61].

The processed data reflected a mixture of negative, neutral, and positive sentiments across platforms, as summarized in Table 1. The online review dataset showed substantial variation in the volume and distribution of sentiments between media sources. Of the 573 (11.3%) reviews contributed by Twitter, 218 (38%) were categorized as negative, 251 (43.8%) as neutral, and 104 (18.2%) as positive entries. Of the 282 (5.6%) reviews generated by TikTok, the majority (n=225, 79.5%) were categorized as neutral. For video-based platforms, such as YouTube, which produced 96 (1.9%) reviews, 89 (92.7%) reviews were categorized as neutral.

**Table 1:** Distribution of review data by platform.

Platform	Negative reviews (n=312), n (%)	Neutral reviews (n=4389), n (%)	Positive reviews (n=346), n (%)	Total (N=5047), n (%)
Twitter (X)	218 (69.9)	251 (5.7)	104 (30.1)	573 (11.3)
TikTok	19 (6.1)	225 (5.1)	38 (11.0)	282 (5.6)
Videos	1 (0.3)	89 (2.0)	6 (1.7)	96 (1.9)
News portals	50 (16.0)	2885 (65.7)	105 (30.3)	3040 (60.2)
Podcasts	0	6 (0.1)	0	6 (0.1)
Forums	0	5 (0.1)	0	5 (0.1)
Blogs	11 (3.5)	329 (7.5)	13 (3.8)	353 (7.0)
Websites	13 (4.2)	599 (13.6)	80 (23.1)	692 (13.7)

News platforms exhibited the largest volume of data, amounting to 3040 (60.2%) reviews; however, most were neutral (n=2885, 94.9%), accompanied by 105 (3.5%) positive and 50 (1.6%) negative sentiments. Blogs and websites contributed 353 (7%) and 692 (11.3%) reviews, respectively, with neutral sentiment dominating both categories (n=329, 93.2%, for blogs and n=599, 86.6%, for websites).

Overall, these findings indicated substantial cross-platform variation in sentiment distribution. Despite differences in volume, most platforms show a predominance of neutral sentiment, suggesting that public discourse on specialist medical education tends to be descriptive or informational rather than overtly evaluative.

These data show that Twitter has the highest proportion of negative sentiment, whereas TikTok has the highest proportion of positive sentiment relative to its volume. Meanwhile, news portals are overwhelmingly neutral, reflecting the reporting style of professional media outlets. Podcasts and forum platforms do not show any negative or positive sentiment, while video and web-based platforms have a low number of negative- and positive-sentiment reviews. Furthermore, news platforms show a fairly low number of negative- and positive-sentiment reviews, with neutral sentiment dominating. Overall, the percentage of negative sentiment of around 6.2% (n=312 reviews) is smaller than the percentage of positive sentiment of 6.9% (n=346 reviews).

**Figure 3:**
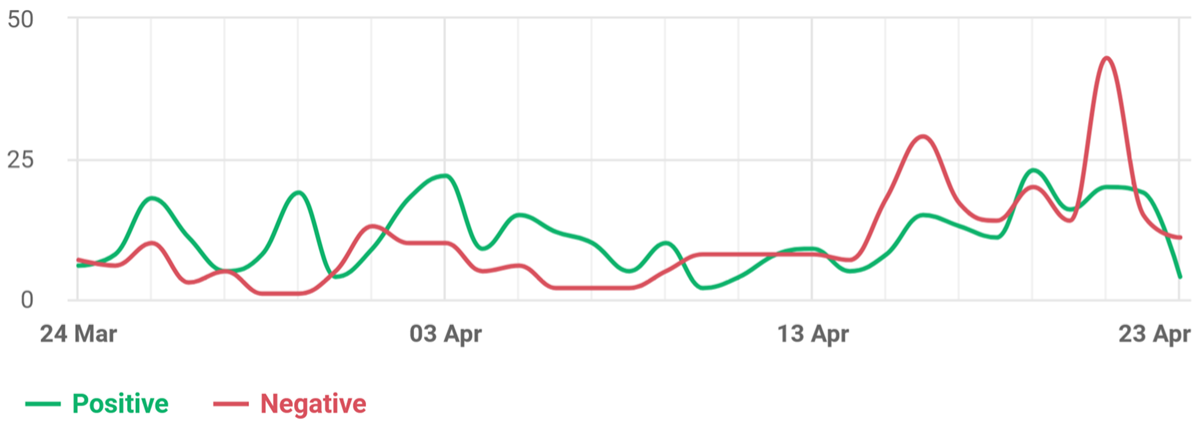
Sentiment-based review history for 1 month.

Figure 3 shows the trend in daily positive and negative reviews for 1 month. At the beginning of the month, the number of positive and negative reviews was relatively low and stable. However, starting April 16, there was a significant increase in the number of positive and negative reviews. This is in line with reports in the mass media regarding the results of PPDS mental health screening [16] on that date [12], which may have triggered an increase in online activity related to the topics discussed in the research.

### Coding Review Topics

After carrying out data cleaning, 292 (5.8%) reviews with negative sentiment and 317 (6.3%) reviews with positive sentiment were obtained. These data were then analyzed using NVivo 14.

#### Negative-Review Topic Coding

As the initial aim of this research, each sentiment in the review was coded to find review themes. We used the *autocode* feature provided by NVivo 14 to automatically recognize and mark text or other qualitative data based on predetermined rules. NVivo applies techniques such as text analysis and content analysis to identify patterns in text and tag or code data accordingly [62].

To ensure the credibility of the coding process, the results of autocode generation were followed by manual validation carried out by two independent coders. An intercoder agreement check was conducted, and discrepancies were resolved through discussion to strengthen the reliability of the coding.

The autocode results produced 28 negative-review topic codes, as presented in Table 2. To improve interpretability, these topics were subsequently grouped into six broader clusters: *medical workforce*, *educational system and training*, *health care institutions and patients*, *financial and resource constraints*, *media and public discourse*, and *other student life factors*.

The largest cluster was “educational system and training” (n=8, 28.6%, topics), followed by “medical workforce” (n=7, 25%, topics). This highlights that criticisms are not only about individuals (doctors, students, residents) but also about structural issues in programs and curricula. Smaller clusters, such as “media and public discourse” and “student life factors,” capture contextual influences that extend beyond institutional boundaries, underscoring the multifaceted nature of the challenges.

**Figure 4:**
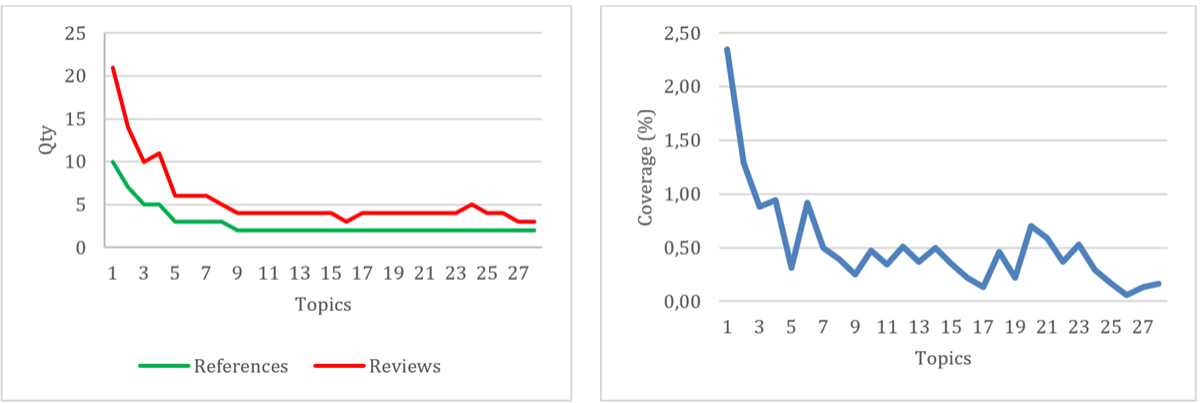
References and reviews (a) and coverage (b) for each negative-review topic.

Discussion topic data generated by NVivo 14 related to negative community reviews revealed a variety of topics highlighted in these reviews. Of the 28 topics identified, based on the number of references and reviews (Figure 4a), the data showed that the topics of discussion tended to be spread out, with significant variations in the number of references and reviews. Most topics had an equal number of references and reviews, with some topics, such as “doctors” and “specialists,” having a slight difference in the number of references and reviews. However, when looking from a coverage perspective (Figure 4b), most topics had low scores, indicating that these topics were not widely discussed in the data. Nevertheless, it was still important to pay attention to them because some topics could affect the validity and representativeness of analysis results.

**Table 2:** Topics in negative reviews.

Topic number	Topic	Topic feature words	Cluster
1	Doctors	intern doctors, resident doctors, alone specialist doctors, emergency room doctors, previous doctors, procuring specialist doctors, prospective doctors, teachers, specialist doctors	Medical workforce
2	Specialists	lung specialist, alone specialist doctors, dermatology specialists, otolaryngology specialists, procuring specialist doctors, teachers, specialist doctors	Medical workforce
3	Students	medical student status, boarding student, dental students, nursing students	Educational system and training
4	Hospitals	vertical hospitals, hospital environment, hospital management, large hospitals	Health care institutions and patients
5	Teaching	teaching staff, teachers, specialist doctors	Medical workforce
6	Medicine	internal medicine, traditional medicine	Educational system and training
7	Programs	8th pregnancy program, expert master education program, study program	Educational system and training
8	Lungs	lung specialist, lung disease	Medical workforce
9	Nursing	nursing student, senior nurse	Medical workforce
10	Patients	sleep patients, trigger patients	Health care institutions and patients
11	Sleep	patient sleep, sleeping pills	Student life factors
12	Media	media briefings, social media	Media and public discourse
13	Vertical hospitals	vertical hospitals	Health care institutions and patients
14	Health	community health center, first-class health clinic	Health care institutions and patients
15	Education	expert master education program, medical education providers	Educational system and training
16	Expert master	expert master candidates, expert master education program	Educational system and training
17	Suitable	following suit, really suitable	Student life factors
18	Money	received money, scientific money	Financial and resource constraints
19	Medical student status	medical student status	Educational system and training
20	Internal doctors	internal doctors	Medical workforce
21	Results	good results, solid results	Educational system and training
22	Community	community health center, local community	Health care institutions and patients
23	Working	work strikes, working hours	Financial and resource constraints
24	Resident doctors	resident doctor	Medical workforce
25	Urine	urine bag, urine hunter	Financial and resource constraints
26	Smells	formalin smell, n't smell	Financial and resource constraints
27	Stages	stage compositor, stage rotation	Educational system and training
28	House	boarding house, halfway house	Financial and resource constraints

#### Positive-Review Topic Coding

Autocoding carried out by NVivo 14 on positive reviews produced 17 topic codes, as summarized in Table 3.

**Figure 5:**
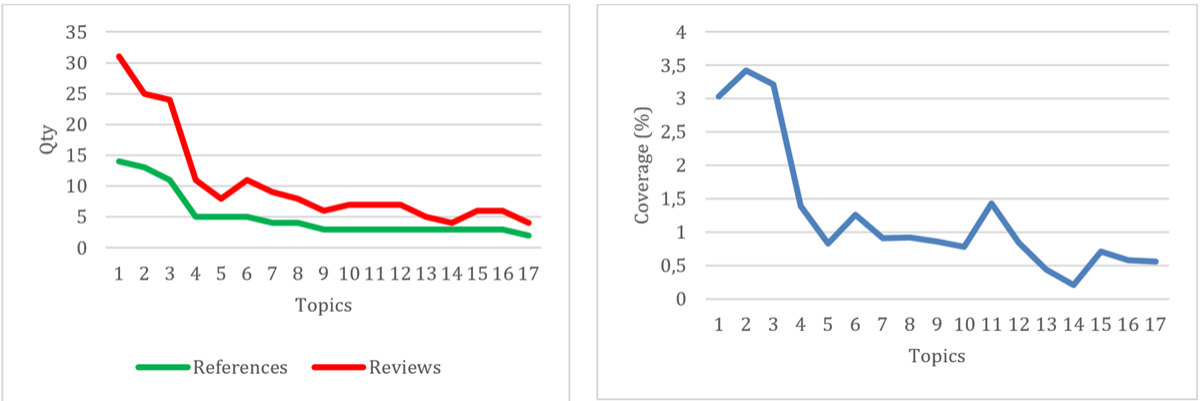
References and reviews (a) and coverage (b) for each positive-review topic.

Based on the number of references and reviews (Figure 5a), the data showed significant variations in the number of references and reviews for each topic. Most topics had an equal number of references and reviews, but some had quite striking differences, such as the topics “doctors” and “services,” which had a higher number of reviews than the number of references. However, from a coverage perspective (Figure 5b), the majority of topics had quite high scores, indicating that these topics were widely discussed in the data. Topics such as “doctors” and “services” even had coverage values above 3% (17 reviews), indicating that they are the main focus of positive community reviews. Even though some topics had lower coverage values, the majority of them were still quite high, showing the variety of positive topics discussed in public conversations.

**Table 3:** Topics in positive reviews.

Topic number	Topic	Topic feature words	Operational definition
1	Doctors	professional doctor candidates, resident doctors, aspiring doctors, general doctors, eye specialists, online doctor apps	Mentions of doctors in a positive sense, reflecting professionalism, expertise, or accessibility
2	Services	superior service, pediatrician services, integrated services, rehab services	References to the quality, diversity, or efficiency of health care services
3	Students	medical students, students	Positive perception of medical students’ role, contribution, or progress
4	Health	various health problems, health centers, health fields, health practitioners	General statements about health promotion, prevention, or community health benefits
5	Surgery	cardiac, cataract, orthopedic, preventive surgery	Praise for successful surgical procedures or advanced surgical options
6	Specialists	pediatric specialists, beauty specialists, eye specialists	Recognition of specialist expertise or availability in various fields
7	Treatment	beauty treatment, canal treatment, treating skin, treatment techniques	Appreciation of effective or innovative treatment modalities
8	Practitioners	general practitioner, health practitioner, health care practitioner	Positive portrayal of practitioners’ skills and dedication
9	Facilities	equivalent facilities, sophisticated facilities, surgical facilities	Highlighting the adequacy, modernity, or comfort of health care facilities
10	Consultation	direct consultation, online doctor consultation application, psychological consultation services	Positive feedback on ease of access and quality of medical consultations
11	Superior service	superior service	Reinforcement of outstanding service quality in medical practice
12	Fields	health fields, medical fields, various fields	References to the diversity of medical and health disciplines
13	Beauty	beauty specialist, beauty treatment, well-maintained beauty	Positive associations with medical aesthetics and wellness
14	Rooms	physiotherapy rooms, reflection rooms, speech therapy rooms	Praise for the availability and functionality of medical or therapeutic rooms
15	Practice	specialist pediatrician practice services, practice schedule	Mentions of structured and accessible medical practices
16	Programs	23 study programs, male pregnancy program, professional program	Positive recognition of medical or educational programs
17	Choices	menu choices, the right choice	Appreciation of having diverse and appropriate health care or program options

### Cognitive Perspectives and Themes

Cognitive perspectives were established to help explain how society responds to the Icarus Paradox and how these perceptions influence their behavior regarding medical education. The cognitive perspective in the research context refers to the way individuals understand, interpret, and give meaning to information obtained from their environment. This includes how people process the information they obtain from online media, such as news articles, comments on social media, blogs, and discussion forums, as well as how their views are formed, influenced, and expressed through these platforms [27]. The combination of all topics in positive and negative reviews is then sorted to develop appropriate themes. Each corresponding theme is then grouped into a cognitive perspective. Based on all the reviews collected in this research, we defined four cognitive perspectives: *education system*, *policy*, *society’s views of students*, and *health care services* (Table 4).

**Table 4:** Cognitive perspectives and themes by review topic.

Cognitive perspective and themes		Negative topics	Positive topics
**Education system**			
	Professional challenges	Topic 1: doctors Topic 24: resident doctors Topic 25: urine	Topic 1: doctors Topic 12: fields
	Economic challenges	Topic 18: money	—^a^
	Workload	Topic 19: medical student status Topic 20: internal doctors	—
	Medical understanding	Topic 8: lungs	—
	Teacher qualifications	Topic 5: teaching Topic 17: suitable	—
	Medical education	Topic 7: programs Topic 13: vertical hospitals Topic 15: education Topic 16: expert master	Topic 4: health Topic 16: programs
**Policy**			
	Government policy	Topic 23: working Topic 27: stages	—
**Society’s views of students**			
	About students	Topic 3: students Topic 28: houses	Topic 3: students
	Community involvement	Topic 12: media	Topic 6: specialists
**Health care services**			
	Quality of health services	Topic 2: specialists Topic 4: hospitals Topic 6: medicine Topic 9: nursing Topic 14: health Topic 21: results Topic 26: smells	Topic 2: services Topic 5: surgery Topic 8: practitioners Topic 9: facilities Topic 11: superior service Topic 13: beauty Topic 15: practice
	Patient experience	Topic 10: patients Topic 11: sleep Topic 22: community	Topic 7: treatment Topic 10: consultation Topic 14: rooms Topic 17: choices

^a^Not applicable.

The “education system” perspective explains prospective doctors’ direct experience with the medical education system and health facilities, which will shape their perceptions and assessments of the quality and effectiveness of the system. The information they obtain from online media can influence how they interpret and remember their own experiences. This perspective explains several themes, such as professional challenges, economic challenges, workload, medical understanding, teaching qualifications, and medical education. The “policy” perspective explains the public’s response to assessing the quality of medical education and health services, which is linked to the impact of government policy. Information about government policies, societal responses, and changes in the health system may influence the public’s perceptions of the medical education system. This perspective focuses solely on the theme of government policy. Furthermore, the “society’s views of students” perspective influences perceptions of medical education and the medical profession, shaped by the social support individuals receive from their environment. The perspectives of friends, family, and the medical community can influence how individuals understand and respond to the challenges they face in their medical education and careers. This perspective explains themes such as student views, as well as the themes of community engagement and public health. The “health care services” perspective explains public reviews regarding the experience of receiving services and the results obtained when visiting a doctor. This perspective explains themes such as the quality of health services and the patient experience. The entire cognitive perspective, along with its respective themes, is further explained in the coding process of the negative and positive reviews next.

**Figure 6:**
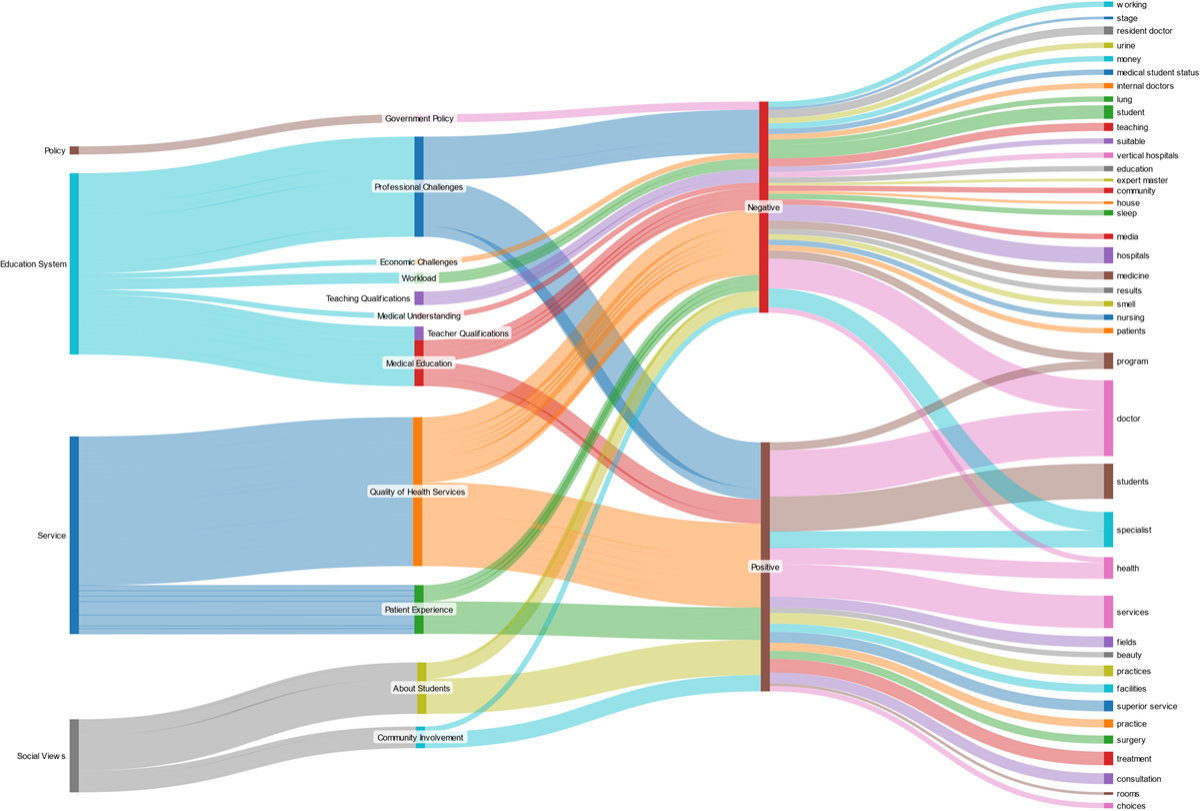
Sankey diagram of topic distribution for cognitive perspectives and themes.

Visualization of topic flow across cognitive perspectives, themes, sentiments, and issues was produced using a Sankey diagram (Figure 6). The diagram shows that five topics (*program*, *doctor*, *student*, *specialist*, and *health*) contained both positive and negative sentiments, indicating contradictory reviews and the presence of paradoxical dynamics. Three topics (*program*, *doctor*, *student*) displayed opposing sentiments within the same theme and perspective, whereas two topics (*specialist* and *health*) exhibited sentiment divergence across different themes or perspectives.

The topic *program* centered on the theme of “medical education” within the “education system” perspective. Positive reviews emphasized the value of educational opportunities, such as clinical clerkships and diverse study programs offered by Indonesia’s medical schools. These were seen as mechanisms to broaden competence and strengthen professional readiness. At the same time, negative sentiment highlighted recurring concerns, including bullying cases in the PPDS and the closure of certain specialties, such as radiology. These contradictory views—progressive ambition versus internal dysfunction—reflect the Icarus Paradox, where efforts to elevate educational excellence may simultaneously generate harmful systemic pressures [35,36].

The topic *doctors* related to “professional challenges” within the “education system” perspective. Positive sentiment acknowledged the achievements of doctors and medical students, recognition of expertise, and optimism surrounding innovations, such as online consultation platforms. However, negative sentiment pointed to structural barriers, such as shortages of specialist lecturers, harsh working conditions, low compensation for interns, and ethical concerns related to patient rights. These tensions illustrate the conflict between professional ideals and the realities imposed by institutional limitations.

The topic *students* was discussed under the “society’s views of students” perspective. Positive sentiment highlighted recognition of student accomplishments, the use of simulators and models in training, and hopes for producing compassionate and resilient physicians. Conversely, negative reviews drew attention to workload imbalance, ethical dilemmas when shifting roles, and perceptions of injustice in training environments. These competing narratives reflect the paradox of aspiring to excellence, while navigating a system that can undermine fairness and well-being.

Sentiment patterns for the topic *specialists* also revealed contrasting perspectives. Positive sentiment, categorized under “community involvement,” underscored the indispensable role of specialists in delivering quality care, especially in underserved areas. Public comments highlighted trust in specialists’ ability to accelerate recovery and address complex conditions. In contrast, negative sentiment, linked to the “health care services” perspective, emphasized limited specialist availability, particularly in remote regions, as well as funding and resource constraints. These shortages translate directly into service gaps, as illustrated by cases in which patients cannot obtain urgently needed specialist care.

Finally, the topic *health* contained both positive and negative sentiment across differing themes. Positive reviews, aligned with the “medical education” theme under the “education system” perspective, highlighted improvements in training quality, innovations in medical research, and the contribution of diverse specialists to system enhancement [63]. Negative sentiment, viewed from the “health care services” perspective, reflected dissatisfaction with access barriers, cost concerns, and perceived shortcomings in primary-level care. Public comments described challenges in securing treatment for family members and skepticism toward provider competence in community clinics.

Collectively, these topic-based findings revealed how paradoxical tensions manifest simultaneously across multiple perspectives in Indonesia’s specialist medical education ecosystem, providing critical insight into structural, educational, and service-related challenges.

### Conceptual Model

**Figure 7:**
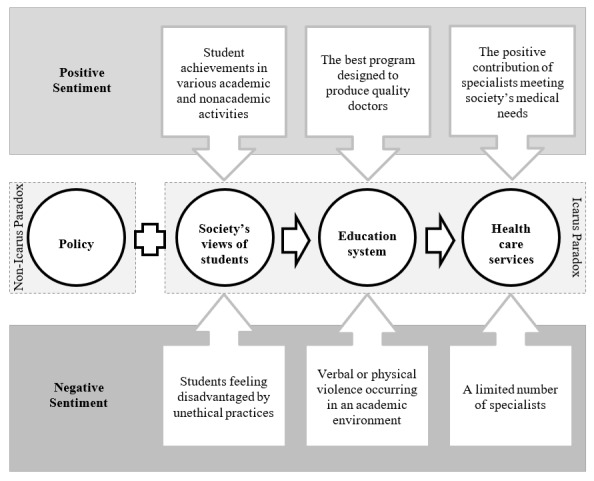
Conceptual model linking the four cognitive perspectives in this study: (1) education system, (2) policy, (3) society’s views of students, and (4) health care services.

Based on the results of our analysis, we proposed a conceptual model that links these four cognitive perspectives. This model can serve as a basis for formulating comprehensive policies and strategies to enhance medical education, improve public perception of the medical profession, and overall health services. With an integrated and holistic approach, it is hoped that the Icarus Paradox identified in this study can be overcome, thereby improving the overall quality of the health system. The proposed conceptual model is shown in Figure 7. Perspectives with white circles have the Icarus Paradox effect, while perspectives marked with gray circles do not have the Icarus Paradox effect. The “education system” perspective in this conceptual model is the primary component in producing quality health services, which is largely determined by the input and role of regulations, as well as “society’s views of students” regarding prospective medical students.

## Discussion

### Principal Findings

This study identified three major cognitive perspectives—education system, health care services, and society’s views of students—frequently reflected in public reviews, while policy issues appeared less prominently. Findings illustrate the Icarus Paradox as it emerges across these perspectives, where ambition and the pursuit of excellence coexist with constraints and systemic limitations. Rooted in classical interpretations of overreach and human limitation [37,38], the paradox becomes visible in how communities describe tensions within Indonesia’s specialist medical education system. In contrast, the policy perspective generated comparatively uniform sentiment, suggesting limited public engagement with regulatory matters.

#### The Icarus Paradox in Specialist Medical Education

The Icarus Paradox describes how attributes that drive success can, when overstretched, evolve into vulnerabilities [7,37,38]. Indonesia’s specialist medical education system reflects this dynamic. Programs such as clinical clerkships and PPDS training offer essential experiential learning and uphold high academic and professional standards [11,44,64]. These strengths, however, may escalate into risks when demands become excessive. Heavy workloads, hierarchical structures, and competitive environments contribute to burnout, intimidation, and uncertainty, exemplified by bullying cases and program closures, such as in radiology [10,11]. Public discourse captured in this study also shows admiration for educational excellence, alongside accounts of program instability and insufficient institutional support, illustrating how the system’s strongest features can simultaneously generate tension.

Addressing these paradoxes requires structural and cultural reforms, including more inclusive learning environments, transparent governance, and stronger mentoring systems grounded in empathy and collaboration [65]. Updating curricula to align with evolving scientific and educational developments is equally vital. Such measures may help sustain academic quality, while minimizing the unintended consequences of overly rigid standards.

#### The Icarus Paradox in the Student Context

Among students, the paradox emerges when determination, resilience, and idealism—traits celebrated at the start of medical training—become sources of vulnerability under excessive pressure. Students often begin their education with strong motivation and aspirations to contribute meaningfully to society [66]. Yet system demands, extended working hours, ethical dilemmas, and limited supervision due to dual lecturer-clinician roles can erode well-being and moral sensitivity [41]. Review data reveal disillusionment when expectations of professional fairness conflict with realities, such as inequitable pathways into practice or nonformal short-course alternatives.

Such tensions reflect how ambition without adequate support can lead to burnout and ethical distress. Strengthening mental health support, promoting fairness in training systems, and integrating ethical guidance are essential to prevent resilience from becoming a liability [65].

#### The Icarus Paradox in the Health Care Context

Paradoxes also materialize within health care delivery [67]. Advances in training, the expansion of simulations, and improvements in health facilities have contributed to better-prepared professionals and broader service coverage. Government initiatives, such as infrastructure development, *Jaminan Kesehatan Nasional* (JKN; National Health Insurance Program) implementation, and adoption of digital health and telemedicine, have improved access in many regions. However, unchecked growth and technological progress can introduce new weaknesses, including practitioner burnout, ethical compromises, or neglect of basic primary care, particularly in underserved communities [8,25].

Persistent disparities between urban and rural areas reflect this tension. Although capacity has increased nationally, specialist-to-population ratios and hospital bed availability remain considerably lower in remote regions [2,68-70]. These gaps demonstrate how reforms designed to elevate care quality can inadvertently reinforce inequality.

Addressing such paradoxes requires integrating community-based training, strengthening support for rural deployment, and ensuring equitable resource allocation [26]. Civil society involvement can help promote fairness and public accountability.

The “education system” perspective illustrates how efforts to sustain high academic standards and broaden learning opportunities in specialist training can inadvertently generate strain. Although diverse programs and research initiatives aim to enhance the quality and relevance of specialist medical education, these strengths may create vulnerabilities when demands exceed student capacity. Heavy academic loads, tight schedules, and competitive selection for placements or specialties can intensify stress and disrupt students’ balance between academic, social, and personal lives. Limited institutional attention to student well-being, insufficient support systems, and cultures that tolerate intimidation or academic mistreatment may further exacerbate these pressures, reinforcing the system’s paradoxical nature.

These dynamics reveal the tension between the ideal of academic excellence and the structural challenges embedded in training environments. Understanding this paradox is essential when evaluating how effectively the educational system prepares future doctors for real-world health care demands.

The “society’s views of students” perspective similarly reflects tensions between public expectations of medical professionalism and students’ lived realities. High academic pressure, limited access to qualified supervisors, financial burdens, and long working hours often conflict with students’ aspirations, contributing to fatigue and reduced well-being. These constraints show how ambition in medical training may collide with structural and ethical limitations, creating conditions consistent with the Icarus Paradox.

Although the “policy” perspective does not directly display paradoxical tension, it remains crucial in mitigating risks emerging from the other three perspectives. Well-designed regulations can support improvements in medical training quality, shape public perceptions of the profession, and promote equitable access to health services.

The “health care services” perspective represents the system’s final output and illustrates tensions between the need for high-quality health care and persistent resource constraints. Limited numbers and uneven distribution of specialists, especially in remote regions, hinder timely access to appropriate care. Funding shortages, inadequate human resources, and complex bureaucratic processes further complicate service delivery. These factors generate a paradox in which aspirations for excellent, equitable care conflict with systemic limitations [67].

Overall, the conceptual model highlights how progress within education, professional expectations, and health care delivery can unintentionally produce new vulnerabilities. Recognizing and managing these paradoxes is essential for creating a more resilient and equitable medical education and health care system.

### Implications

The findings of this study carry significant implications for medical education, health service quality, and the role of specialist medical trainees [26]. First, within medical education, the Icarus Paradox, reflecting tensions between ambition and structural limitations, shapes student experiences and underscores the urgency of curriculum reform. Greater emphasis is needed on clinical skill development [71], communication, and leadership, which are essential for effective future practice. Educational institutions must also enhance academic and emotional support systems to help students manage stressors that arise during training.

Second, gaps in health service quality between urban and rural regions remain substantial. This highlights the need for increased investment in rural health infrastructure and the equitable placement of medical personnel. Elevating service standards nationwide requires consistent quality assurance, adequate staffing, and measures ensuring equal access to care.

Third, specialist medical trainees play a vital role as agents of change within both the education system and the broader health care landscape. Students must be equipped to navigate contemporary medical challenges and encouraged to engage in system improvement. Sufficient institutional support will enable them to contribute effectively to positive transformation in health services.

Fourth, the findings emphasize that oversight and governance in specialist medical education must be strengthened. Historically, weaknesses in program supervision have received attention only after issues were identified by the Ministry of Health. This implies a strong need for more rigorous monitoring by both government and universities.

Fifth, reliance solely on university-based specialist medical training may limit the number and diversity of specialists needed nationwide. Expanding hospital-based specialist medical training is therefore essential. Since the number of hospitals exceeds the number of medical schools, such an expansion could broaden access, improve specialist distribution to underserved regions, and reduce the training burden on universities. Increasing practitioner involvement in specialist medical education would also enrich training quality. Strengthening hospital-based pathways represents an important step toward addressing disparities and enhancing the national medical education system.

Overall, these implications highlight the need for systemic adjustments in medical education and health care delivery. Recognizing the presence of the Icarus Paradox helps identify areas for improvement and guides targeted reforms. Positive aspects, such as the growing role of hospital-based training, should be sustained and expanded, while structural weaknesses, such as inadequate governance and uneven specialist distribution, must be addressed. With joint commitment from the government, health care institutions, academics, and society, Indonesia can build a more inclusive, sustainable, and community-oriented health system [26], ultimately improving medical education and health service outcomes nationwide.

### Limitations and Further Research

Despite offering valuable insights into the Icarus Paradox within medical education and health services, this study has several limitations. First, the findings rely heavily on online review data, which may not represent the full population. Individuals without internet access or those who do not use online review platforms may be underrepresented, and the anonymous nature of such reviews may affect data reliability.

Second, the study primarily captures community perceptions of the medical education system and specialist medical trainees, which introduces potential subjective bias. Different individuals may express varying experiences and expectations, which may influence sentiment data.

Third, this study does not directly measure clinical performance indicators or objective metrics of service quality. The data analyzed are predominantly qualitative and descriptive. To obtain a more comprehensive understanding of health service quality, future research should integrate quantitative measures with qualitative insights.

Further research can address these limitations in several ways. First, studies may incorporate broader respondent groups, including patients [25], medical practitioners, and students, through structured surveys or interviews to enhance representativeness. Second, advanced analytical techniques, such as deeper text mining or longitudinal sentiment tracking, can identify emerging patterns in digital public discourse. Third, expanding the study scope to examine interactions between variables, such as health care access, medical training quality, and patient satisfaction, over extended time periods (beyond 30 days) will enrich understanding.

Finally, empirical testing of the proposed conceptual model can offer deeper insights into the complex dynamics of Indonesia’s health system. Such research can guide policymakers and educational institutions in designing interventions that enhance system effectiveness and mitigate paradoxical tensions. Ultimately, further investigation will contribute to a more comprehensive understanding of strategies needed to strengthen medical education and health care delivery in Indonesia.

### Conclusion

Indonesia’s specialist medical education system continues to face complex challenges that can be understood through the lens of the Icarus Paradox, where strengths that drive success may simultaneously give rise to failure. Ambition, resilience, and rigorous training enable medical students to pursue excellence, yet these same strengths can lead to burnout, intimidation, and psychological strain when not supported by adequate institutional resources. As Eriksson et al [7] describe, such dynamics reflect an “inverted Icarus” pattern in which progress carries inherent vulnerabilities. Prospective specialist medical students therefore stand at a critical juncture, symbolizing both the promise of advancement and the risks embedded in systemic pressure.

Health service quality also reflects this tension. Although national initiatives, such as infrastructure expansion and the JKN universal health coverage program, have increased access, disparities remain significant. For example, although the doctor-to-population ratio in Jakarta is approximately 1:1000, in some rural provinces, such as East Nusa Tenggara, the ratio drops below 1:7000 [72]. These inequalities illustrate how the push to expand services may exceed the capacity to ensure equitable quality of care. In education, national surveys indicate that more than half of PPDS students report mental health distress and excessive workloads, demonstrating that high standards often translate into substantial personal cost.

Addressing this paradox requires coordinated and targeted reforms. At the policy level, priority actions include (1) redistributing specialist doctors through structured incentives for underserved regions; (2) revising PPDS curricula to incorporate mental health resilience, mentorship, and leadership training; (3) expanding investment in simulation-based learning to reduce overreliance on hospital workloads; and (4) strengthening regulations against intimidation and unsafe learning environments.

At the same time, progress is attainable. Initiatives, such as scholarships for PPDS candidates from remote regions, the development of eLearning platforms in medical schools, and improvements in provincial teaching hospitals, indicate ongoing reform efforts. These programs show that although paradoxical tensions persist, Indonesia is gradually moving toward solutions that balance ambition with sustainable practice.

With collaborative efforts involving government, universities, hospitals, and society, the Icarus Paradox in medical education and health services can be mitigated. This will support Indonesia’s movement toward a more inclusive, equitable, and resilient health care system, one in which the aspiration to produce excellent medical professionals does not compromise their well-being or widen disparities in health service delivery.

## Abbreviations

JKN: Jaminan Kesehatan Nasional (National Health Insurance Program)
PPDS: *Program Pendidikan Dokter Spesialis* (Specialist Doctor Education Program)

## References

[1] Sulphey MM (2020). How Icarus paradox doomed Kingfisher Airlines. Vision: J Bus Perspect.

[2] Mahendradhata Y, Trisnantoro L, Listyadewi S, Soewondo P, Marthias T, Harimurti P, Prawira J, Hort K, Patcharanarumol W (2017). The Republic of Indonesia health system review. Health Systems in Transition, Vol-7, Number-1.

[3] Wallin G (2024). An introduction to R and Python for data analysis: a side-by-side approach. Am Stat.

[4] Bondestam F, Lundqvist M (2020). Sexual harassment in higher education – a systematic review. Eur J High Educ.

[5] Heffernan T, Bosetti L (2021). Incivility: the new type of bullying in higher education. Cambridge J Educ.

[6] Yousaf R, Schmiede R (2016). Harassment Act implementation in higher education institutions. Open J Leadersh.

[7] Eriksson K, Lakomaa E, Nykvist R, Sandström C (2024). Introducing the inverted Icarus paradox in business history – evidence from David and Goliath in the Swedish telecommunications industry 1981–1990. Bus Hist.

[8] Desveaux L (2025). Rethinking healthcare: why paradox science is core to the future of health and health leadership. JHL.

[9] Smith WK, Lewis MW (2011). Toward a theory of paradox: a dynamic equilibrium model of organizing. Acad Manag Rev.

[10] Lili R, Molodynski A, Farrell SM, Citraningtyas T, Kloping NA (2022). Wellbeing and mental health among medical students in Indonesia: a descriptive study. Int J Soc Psychiatry.

[11] Dyah Perwitasari V, Hidayat R (2024). Depression level among neurology resident doctors in the Faculty of Medicine, Universitas Indonesia. Acta Neurol Indones.

[12] Arlinta Deonisia (2024). Depresi, 3,3 Persen Calon Dokter Spesialis Ingin Akhiri Hidup atau Lukai Diri. Kompas.

[13] (2024). Kesaksian calon dokter spesialis yang sempat berusaha bunuh diri – “perundungan dijustifikasi atas nama pendidikan mental. BBC News Indonesia.

[14] (2024). 2.716 Calon dokter spesialis mengalami gejala depresi, 3,3% ingin akhiri hidup. Metro TV News.

[15] Widayanti AW, Green JA, Heydon S, Norris P (2020). Health-seeking behavior of people in Indonesia: a narrative review. J Epidemiol Glob Health.

[16] Martina Ws Nasrun (2024). Depression Phenomenon in PPDS, Fact or Mirage?. Kompas.

[17] Ranasinghe PD, Owusu JT, Bertram A, Michtalik H, Yeh H, Cofrancesco J, Levine D, Miller Iii ER, Marinopoulos S (2022). Depressive symptoms and burnout among medical students: a prospective study. J Gen Intern Med.

[18] Doe S, Coutinho AJ, Weidner A, Cheng Y, Sanders K, Bazemore AW, Phillips RL, Peterson L (2024). Prevalence and predictors of burnout among resident family physicians. Fam Med.

[19] Shalaby R, Oluwasina F, Eboreime E, El Gindi H, Agyapong B, Hrabok M, Dhanoa S, Kim E, Nwachukwu I, Abba-Aji A, Li D, Agyapong VIO (2023). Burnout among residents: prevalence and predictors of depersonalization, emotional exhaustion and professional unfulfillment among resident doctors in Canada. Int J Environ Res Public Health.

[20] Obeng Nkrumah S, Adu MK, Agyapong B, da Luz Dias R, Agyapong VIO (2025). Prevalence and correlates of depression, anxiety, and burnout among physicians and postgraduate medical trainees: a scoping review of recent literature. Front Public Health.

[21] Faghihzadeh E, Eghtesad A, Fawad M, Xu X (2025). Exploring connections between mental health, burnout, and academic factors among medical students at an Iranian University: cross-sectional questionnaire study. JMIR Med Educ.

[22] Lee KP, Yeung N, Wong C, Yip B, Luk LHF, Wong S (2020). Prevalence of medical students' burnout and its associated demographics and lifestyle factors in Hong Kong. PLoS One.

[23] Thamissarakul S, Hongkan W, Wannapaschaiyong P (2024). Factors associated with burnout syndrome among clinical medical students at Chonburi Hospital, Thailand. J Health Sci Med Res.

[24] (2019). Caring for the mental health of the medical workforce. British Medical Association.

[25] Berger S, Saut AM, Berssaneti FT (2020). Using patient feedback to drive quality improvement in hospitals: a qualitative study. BMJ Open.

[26] Bismantara H, Ahern S, Teede HJ, Liew D (2022). Academic health science centre models across the developing countries and lessons for implementation in Indonesia: a scoping review. BMJ Open.

[27] Abid A, Harrigan P, Roy S (2020). A relationship marketing orientation in politics: young voters’ perceptions of political brands’ use of social media. Journal of Strategic Marketing.

[28] Ashraf MA, Khan MN, Chohan SR, Khan M, Rafique W, Farid MF, Khan AU (2021). Social media improves students’ academic performance: exploring the role of social media adoption in the open learning environment among international medical students in China. Healthcare (Basel).

[29] Noya FC, Carr SE, Thompson SC (2023). Attracting, recruiting, and retaining medical workforce: a case study in a remote province of Indonesia. Int J Environ Res Public Health.

[30] Schad J, Lewis MW, Raisch S, Smith WK (2016). Paradox research in management science: looking back to move forward. Acad Manag Ann.

[31] West J, Gallagher S (2006). Challenges of open innovation: the paradox of firm investment in open-source software. R D Manag.

[32] Oliveira P, e Cunha MP (2021). Centralized decentralization, or distributed leadership as paradox: the case of the Patient Innovation’s COVID-19 portal. J Chang Manag.

[33] Klein SP, Spieth P, Söllner M (2024). Employee acceptance of digital transformation strategies: a paradox perspective. J Prodt Innov Manag.

[34] Cunha MPE, Putnam LL (2017). Paradox theory and the paradox of success. Strateg Organ.

[35] Miller D (1992). The Icarus paradox: how exceptional companies bring about their own downfall. Bus Horizons.

[36] Dowds B (2018). Depression and the Erosion of the Self in Late Modernity.

[37] Sherrer D, Franklin A, Kimatian S, Black I, Tsai M (2023). The Icarus paradox and the future of anesthesiology. Anesth Analg.

[38] Biçer C (2021). The Icarus paradox in management: how to be a well-balanced leader?. Nevşehir Hacı Bektaş Veli Üniversitesi SBE Derg.

[39] Vermeulen F (2009). Businesses and the Icarus paradox. Harvard Business Review.

[40] Zreik M (2024). The paradox of educational inequality in Indonesia: socioeconomic implications and paths towards inclusion. Socio-Economic Implic Glob Educ Inequalities.

[41] Winzer R, Lindberg L, Guldbrandsson K, Sidorchuk A (2018). Effects of mental health interventions for students in higher education are sustainable over time: a systematic review and meta-analysis of randomized controlled trials. PeerJ.

[42] Rotenstein LS, Ramos MA, Torre M, Segal JB, Peluso MJ, Guille C, Sen S, Mata DA (2016). Prevalence of depression, depressive symptoms, and suicidal ideation among medical students: a systematic review and meta-analysis. JAMA.

[43] Shapiro J, Decety J (2011). The paradox of teaching empathy in medical education. Empathy: From Bench to Bedside.

[44] Brown T, Williams B, McKenna L, Palermo C, McCall L, Roller L, Hewitt L, Molloy L, Baird M, Aldabah L (2011). Practice education learning environments: the mismatch between perceived and preferred expectations of undergraduate health science students. Nurse Educ Today.

[45] Tess PA (2013). The role of social media in higher education classes (real and virtual) – a literature review. Comput Hum Behav.

[46] Sivakumar A, Jayasingh S, Shaik S (2023). Social media influence on students’ knowledge sharing and learning: an empirical study. Educ Sci.

[47] Wilson C, McDarby V (2023). Social media and mental health. Clin Child Psychol Psychiatry.

[48] Firdos S, Almulla S, Aldossary S, Al Hassan S, Aldhaif L (2023). Exploring the attitudes of medical students towards social media and e-professionalism in Al-Ahsa, Saudi Arabia. Cureus.

[49] Terry K, Yang F, Yao Q, Liu C (2023). The role of social media in public health crises caused by infectious disease: a scoping review. BMJ Glob Health.

[50] Binsar F, Arief M, Tjhin VU, Susilowati I (2025). Exploring consumer sentiments in telemedicine and telehealth services: towards an integrated framework for innovation. J Open Innov Technol Mark Complex.

[51] Kim D, Jung W, Jiang T, Zhu Y (2023). An exploratory study of medical journal’s Twitter use: metadata, networks, and content analyses. J Med Internet Res.

[52] Creswell JW, Creswell JD, Felts DC (2018). Research Design: Qualitative, Quantitative, and Mixed Methods Approaches. Fifth Edition.

[53] Hutagalung SS, Kartika T, Suciska W (2023). Media monitoring analysis of government image in infrastructure development in Indonesia. J Komun.

[54] Hadi SP, Ibrahim MH, Prabawani B, Hamdani RS (2021). Environmental dimension of pandemic COVID-19: case studies of Indonesia. IOP Conf Ser Earth Environ Sci.

[55] Punziano G, De Falco CC, Trezza D (2023). Digital mixed content analysis for the study of digital platform social data: an illustration from the analysis of COVID-19 risk perception in the Italian Twittersphere. J Mix Methods Res.

[56] Chhabra J, Pilkington V, Benakovic R, Wilson MJ, La Sala L, Seidler Z (2025). Social media and youth mental health: scoping review of platform and policy recommendations. J Med Internet Res.

[57] Zhou Z, Jin D, He J, Zhou S, Wu J, Wang S, Zhang Y, Feng T (2024). Digital health platform for improving the effect of the active health management of chronic diseases in the community: mixed methods exploratory study. J Med Internet Res.

[58] Binsar F, Mauritsius T (2020). Mining of social media on Covid-19 big data infodemic in Indonesia. J Comput Sci.

[59] Lee YU, Chung SH, Park JY (2024). Online review analysis from a customer behavior observation perspective for product development. Sustainability.

[60] Limna P (2023). The impact of NVivo in qualitative research: perspectives from graduate students. J Appl Learn Teach.

[61] Dereń K, Rajda K (2025). How to master AI-powered sentiment analysis in 2025?. Brand24.

[62] Jackson K, Bazeley P (2019). Qualitative Data Analysis with NVivo. 3rd Edition.

[63] Binsar F, Legowo N (2020). Design of cloud computing outpatient registration model through SMS messages at hospitals using TOGAF ADM. Int J Recent Technol Eng.

[64] Binsar F, Kartono R, Bandur A, Kosasih W (2022). Digital transformation of information fulfillment and patient engagement for health service safety.

[65] Mustika R, Nishigori H, Ronokusumo S, Scherpbier A (2019). The odyssey of medical education in Indonesia. Asia Pacific Sch.

[66] Zechariah S, Ansa BE, Johnson SW, Gates AM, Leo GD (2019). Interprofessional education and collaboration in healthcare: an exploratory study of the perspectives of medical students in the United States. Healthcare (Basel).

[67] Hofmann B (2001). The paradox of health care. Health Care Anal.

[68] Sutrisno MD (2023). Shortages of medical doctors in indonesia, is it true?. Asian J Heal Res.

[69] Risky YPA, Projo NWK (2022). The effect of the digital economy on indecent work in Indonesia 2019.

[70] Awofeso N, Rammohan A, Asmaripa A (2012). Exploring Indonesia’s “low hospital bed utilization-low bed occupancy-high disease burden” paradox. J Hosp Adm.

[71] Binsar F, Riantono I, Kartono R, Bandur A, Kosasih W (2023). Gamification to grow motivation for interactive engagement of health nurses in using health information systems: a conceptual framework.

[72] Putri LP, Russell DJ, O'Sullivan BG, Kippen R (2021). Factors associated with working in remote Indonesia: a national cross-sectional study of early-career doctors. Front Med (Lausanne).

